# Editorial: The Reciprocal Relationship Between Sleep and Stress in Elite Athletes

**DOI:** 10.3389/fpsyg.2021.797847

**Published:** 2021-11-22

**Authors:** Jacopo A. Vitale, Nedelec Mathieu, Skorski Sabrina, Lastella Michele

**Affiliations:** ^1^IRCCS Istituto Ortopedico Galeazzi, Milan, Italy; ^2^French National Institute of Sport (INSEP), Paris, France; ^3^Institute of Sport and Preventive Medicine, Saarland University, Saarbrücken, Germany; ^4^Appleton Institute for Behavioral Science, Central Queensland University, Rockhampton, QLD, Australia

**Keywords:** sleep, REM, PSG, actigraphy, recovery, training, competition, well-being

Modern elite athletes are facing more mental, emotional and social demands than ever before, including, amongst others, pressure on personal relationships, media demands, sponsor needs and public interest (Walsh et al., 2021). The majority of elite athletes sleep less than the recommended night-time sleep duration of 7–9 h per night and obtain less than their self-assessed sleep need (Sargent et al., 2021). Many endogenous and environmental factors are able to negatively influence sleep duration and quality in athletes, including evening high-intensity training, pre-competition anxiety, mental fatigue or long-haul travel (Aloulou et al., 2021; Janse van Rensburg et al., 2021; Walsh et al., 2021). Unfortunately, poor sleep can have negative effects on both psychological and physiological aspects which can ultimately lead to an impairment of physical and mental performance (Fullagar et al., 2015; Filipas et al., 2021; Vitale et al., 2021). Thus, it was the aim of this Research Topic to explore the reciprocal relationship between sleep and stress, and to provide insights into potential contributions regarding athletic performance and health.

## Sleep and Stress: What Is the Link?

The relationship between stress and sleep is complex and bi-directional. Sleep is crucial for preserving emotional regulation and functional magnetic resonance imaging studies showed alterations in emotional brain networks in sleep deprived humans, especially in the limbic system. On the contrary, previous studies demonstrated that stress has a negative effect on sleep and conversely many emotions, such as sadness, shame, guilt, or anger have been associated with poor sleep (Vandekerckhove and Cluydts, 2010; Kahn et al., 2013). The psycho-socio-physiological stresses placed on modern elite athletes may actually result in an inability to obtain appropriate sleep and, since very few studies have been conducted until now, the sleep-stress relationship warrants further attention in sport settings too.

Hrozanova et al. evaluated the association between sleep, mental strain, and training load in junior endurance athletes and they observed that higher levels of mental strain were associated both with a reduction in sleep efficiency and sleep duration. These results are in line with previous papers showing the negative effect of psychological stress on sleep in the general population (Akerstedt et al., 2007) but the present study extends these findings to the young athletic population (Nédélec et al., 2021). The same authors highlighted, in a second paper, differences between athletes based on their reactions to stress and sleep quality: one group of athletes, scored as good sleepers on the Pittsburgh Sleep Quality Index (PSQI), had facilitative reactions to stress and satisfactory sleep quality, whereas the other group, composed by poor sleepers based on PSQI scores, exhibited maladaptive reactions to stress and inadequate sleep quality. That said, it is important to highlight that all athletes understood that optimal sleep was important for their functioning and stress levels (Hrozanova et al.).

## Other Factors Contributing to Sleep Complaints and First-Night Effect in Athletes

de Blasiis et al. investigated the relationship between sleep pattern and possible stressors in elite athletes through a mixed-method approach and the results showed that training load and the sleep environment (i.e. noise, temperature and mattress quality) negatively influenced athletes' sleep. Further, student athletes had reduced sleep duration, delayed bedtime and early get-up time compared with non-student athletes. The authors concluded that the study requirements negatively impacted student athletes' sleep and this finding is in line with previous studies reporting that late bedtime, caused by social activities and lifestyle, combined with early study start times, represents a challenge to achieve the recommended sleep duration in adolescents (Crowley et al., 2018).

Further, for the first time in a sport-specific setting, Hof zum Berge et al. tested, the “first-night effect” in a sample of elite athletes utilizing a portable polysomnography (PSG). Athletes were required not to modify their usual training and sleeping schedules for the 2 days of monitoring and the PSG data highlighted a difference between the first and the second night in non-REM sleep, total wake time, sleep efficiency and wake percentage and N3 sleep onset latency. Although there are limitations related to short-term sleep monitoring, these findings underline the importance of the first-night effect during a PSG monitoring in home-based setting for athletes. Sport experts and researchers need to develop a greater understanding of the issues that may disturb sleep in athletes and appropriate assessment tools for sleep to make the best possible decisions.

## “Mind-Based” Solutions for Athletes' Stress and Sleep

Given that many factors are able to negatively affect athletes' sleep and psychological stress is one of the most influential factors within this population, it is necessary to find effective solutions targeting athletes' mental health. Li et al. evaluated the effect of a brief mindfulness induction on university athletes' sleep following evening training. Prior to sleep, a group of athletes received a self-administered 6 min mindfulness induction through a video clip and sleep, training and mood state variables were collected. A reduction in pre-sleep arousal and increased level of overall rest and sleep quality, but not duration, were observed. In addition, pre-sleep arousal was found to be a mediator in the relationship between the brief mindfulness induction and sleep. Similarly, Jones et al. investigated the effect of an 8 weeks course of a mindfulness-based stress reduction on psychological well-being, subjective and objective sleep parameters and physical performance in a sample of NCAA Division female rowers. The course consisted of class sessions that lasted 75 min, once per week, and led by a professional instructor. The results showed an improvement in psychological well-being, sleep quality and also the physical performance, assessed by a 6k ergometer test, improved. These two studies highlighted, from a practical point of view, that different mindfulness-based approaches can be used as an effective way to enhance athletes' mental health and overall sleep quality, both in acute (i.e., after high-intensity night training) or chronic (i.e., during the competitive season) conditions.

In conclusion, the balance between sleep and stress is delicate. Thus, athlete support staff need to avoid the “vicious cycle” in which poor sleep may compromise emotional regulation, increase negative emotion, which may in turn disrupt sleep.

## Author Contributions

JV wrote the first of draft of the present manuscript. All authors listed have made a substantial and direct, and intellectual contribution to the work and approved it for publication.

## Funding

This work was supported by the Italian Ministry of Health (Ricerca Corrente).

## Conflict of Interest

The authors declare that the research was conducted in the absence of any commercial or financial relationships that could be construed as a potential conflict of interest.

## Publisher's Note

All claims expressed in this article are solely those of the authors and do not necessarily represent those of their affiliated organizations, or those of the publisher, the editors and the reviewers. Any product that may be evaluated in this article, or claim that may be made by its manufacturer, is not guaranteed or endorsed by the publisher.
